# Modifiable risk factors and long term risk of type 2 diabetes among individuals with a history of gestational diabetes mellitus: prospective cohort study

**DOI:** 10.1136/bmj-2022-070312

**Published:** 2022-09-21

**Authors:** Jiaxi Yang, Frank Qian, Jorge E Chavarro, Sylvia H Ley, Deirdre K Tobias, Edwina Yeung, Stefanie N Hinkle, Wei Bao, Mengying Li, Aiyi Liu, James L Mills, Qi Sun, Walter C Willett, Frank B Hu, Cuilin Zhang

**Affiliations:** 1 Global Centre for Asian Women’s Health, Yong Loo Lin School of Medicine, National University of Singapore, Singapore; 2 Department of Obstetrics and Gynaecology, Yong Loo Lin School of Medicine, National University of Singapore, Singapore; 3 Bia-Echo Asia Centre for Reproductive Longevity and Equality, Yong Loo Lin School of Medicine, National University of Singapore, Singapore; 4 Department of Epidemiology, Harvard TH Chan School of Public Health, Boston, MA, USA; 5 Department of Nutrition, Harvard TH Chan School of Public Health, Boston, MA, USA; 6 Department of Medicine, Beth Israel Deaconess Medical Center, Harvard Medical School, Boston, MA, USA; 7 Channing Division of Network Medicine, Brigham and Women’s Hospital and Harvard Medical School, Boston, MA, USA; 8 Department of Epidemiology, Tulane University School of Public Health and Tropical Medicine, New Orleans, LA, USA; 9 Division of Preventive Medicine, Department of Medicine, Brigham and Women’s Hospital and Harvard Medical School, Boston, MA, USA; 10 Epidemiology Branch, Division of Population Health Research, Eunice Kennedy Shriver National Institute of Child Health and Human Development, National Institutes of Health, Rockville, MD, USA; 11 Department of Biostatistics, Epidemiology and Informatics, Perelman School of Medicine, University of Pennsylvania, Philadelphia, PA, USA; 12 Division of Life Sciences and Medicine, University of Science and Technology of China, Hefei, China; 13 Biostatistics & Bioinformatics Branch, Division of Population Health Research, Eunice Kennedy Shriver National Institute of Child Health and Human Development, National Institutes of Health, Rockville, MD, USA

## Abstract

**Objectives:**

To evaluate the individual and combined associations of five modifiable risk factors with risk of type 2 diabetes among women with a history of gestational diabetes mellitus and examine whether these associations differ by obesity and genetic predisposition to type 2 diabetes.

**Design:**

Prospective cohort study.

**Setting:**

Nurses’ Health Study II, US.

**Participants:**

4275 women with a history of gestational diabetes mellitus, with repeated measurements of weight and lifestyle factors and followed up between 1991 and 2009.

**Main outcome measure:**

Self-reported, clinically diagnosed type 2 diabetes. Five modifiable risk factors were assessed, including not being overweight or obese (body mass index <25.0), high quality diet (top two fifthsof the modified Alternate Healthy Eating Index), regular exercise (≥150 min/week of moderate intensity or ≥75 min/week of vigorous intensity), moderate alcohol consumption (5.0-14.9 g/day), and no current smoking. Genetic susceptibility for type 2 diabetes was characterised by a genetic risk score based on 59 single nucleotide polymorphisms associated with type 2 diabetes in a subset of participants (n=1372).

**Results:**

Over a median 27.9 years of follow-up, 924 women developed type 2 diabetes. Compared with participants who did not have optimal levels of any of the risk factors for the development of type 2 diabetes, those who had optimal levels of all five factors had >90% lower risk of the disorder. Hazard ratios of type 2 diabetes for those with one, two, three, four, and five optimal levels of modifiable factors compared with none was 0.94 (95% confidence interval 0.59 to 1.49), 0.61 (0.38 to 0.96), 0.32 (0.20 to 0.51), 0.15 (0.09 to 0.26), and 0.08 (0.03 to 0.23), respectively (P_trend_<0.001). The inverse association of the number of optimal modifiable factors with risk of type 2 diabetes was seen even in participants who were overweight/obese or with higher genetic susceptibility (P_trend_<0.001). Among women with body mass index ≥25 (n=2227), the hazard ratio for achieving optimal levels of all the other four risk factors was 0.40 (95% confidence interval 0.18 to 0.91). Among women with higher genetic susceptibility, the hazard ratio of developing type 2 diabetes for having four optimal factors was 0.11 (0.04 to 0.29); in the group with optimal levels of all five factors, no type 2 diabetes events were observed.

**Conclusions:**

Among women with a history of gestational diabetes mellitus, each additional optimal modifiable factor was associated with an incrementally lower risk of type 2 diabetes. These associations were seen even among individuals who were overweight/obese or were at greater genetic susceptibility.

## Introduction

Type 2 diabetes arises from a combination of lifestyle and genetic factors. Previous clinical trials support the effectiveness of lifestyle on preventing the disorder.^1^ ^2^ Observational evidence shows that up to 90% of cases might be prevented or delayed by maintaining a healthy weight and adopting a healthy lifestyle.^3^ ^4^ Furthermore, a healthy lifestyle could partially mitigate the excess risk owing to underlying genetic susceptibility to type 2 diabetes.^5^ ^6^ ^7^ Despite the ample evidence for the effectiveness of a healthy lifestyle, the disorder remains as a major public health challenge in the US as a result of high obesity prevalence^8^ and low rates of adherence to healthy lifestyle practices.^9^ ^10^ According to the National Diabetes Statistics Report in 2020, 37.1 million (14.7%) US adults aged 18 years or older have diabetes, with type 2 diabetes accounting for 90-95% of cases.^11^ Diagnosis of the disorder is often delayed with metabolic abnormalities preceding the diagnosis by 4-6 years, often leaving patients with potentially irreversible damage to the cardiovascular, renal, and neurological systems.^12^ ^13^ As a result, identifying high risk groups early with effective intervention is key for preventing type 2 diabetes and its related complications.

Women (or people who menstruate) who have developed gestational diabetes mellitus represent one such high risk population of type 2 diabetes. Gestational diabetes mellitus is a pregnancy complication affecting about 8% of pregnancies in the US.^14^ Prevalence of the disorder is rising, potentially due to a combination of growing obesity and increasing maternal age.^15^ ^16^ Compared with the general population, women with a history of gestational diabetes mellitus face up to a 10-fold higher risk of progressing to type 2 diabetes.^17^ Gestational diabetes mellitus unmasks underlying cardiometabolic dysregulation or susceptibility at an early age, offering an opportunity for early intervention to prevent subsequent progression to type 2 diabetes.^18^ ^19^ While several individual diet and lifestyle factors have been related to risk of type 2 diabetes among these high risk women,^20^ ^21^ ^22^ the combined associations of modifiable risk factors on long term risk of the disorder are less well understood. Additionally, whether adherence to optimal levels of modifiable factors would reduce risk of the disorder even among those who are overweight/obese or have a greater genetic susceptibility to type 2 diabetes remains to be elucidated; this association is critical, because the prevalence of being overweight/obesity and genetic susceptibility to type 2 diabetes are higher in women with gestational diabetes mellitus than in the general population.^14^ To fill in these research gaps, we prospectively evaluated the associations of adherence to optimal levels of five modifiable risk factors, including healthy body mass index, high quality diet, regular physical activity, moderate alcohol consumption, and not smoking, with the risk of progression to type 2 diabetes among women with a history of gestational diabetes mellitus from the longitudinal Nurses’ Health Study II (NHS II) with 28 years of follow-up. We also assessed whether these associations were modified by obesity status or underlying genetic susceptibility for type 2 diabetes.

## Methods

### Study population

The study population consisted of women with a history of gestational diabetes mellitus in NHS II, a part of the Diabetes and Women’s Health study for investigating risk factors for type 2 diabetes progression among women with gestational diabetes mellitus.^18^ The NHS II enrolled 116 429 female nurses registered in the US aged 24-44 years when the study was initiated in 1989.^23^ Women were followed up biennially since baseline. The study protocol was approved by the institutional review boards of the Brigham and Women’s Hospital and Harvard T H Chan School of Public Health, with return of the questionnaires implying participant consent.

For the current investigation, follow-up started from 1991—the first follow-up period when NHS II participants reported detailed information on diet. Participants were included in the present study if they reported a history of any pregnancy with complications of gestational diabetes mellitus before 1991. Participants also became eligible later if they reported any incident gestational diabetes mellitus between 1991 and 2001, after which most of the participants had passed reproductive age. A previous validation study in this cohort suggested a high level of testing for gestational diabetes mellitus and high concordance between self-reported disease and disease confirmed via medical records (94%).^24^ We excluded participants if they had reported a history of multiple gestation pregnancy (ie, twins or triplets) or a history of type 2 diabetes, cardiovascular diseases (myocardial infarction or stroke), or cancer (except non-melanoma skin cancer) before the first questionnaire after a gestational diabetes mellitus diagnosis. The final analytical sample consisted of 4275 women with a history of gestational diabetes mellitus (the follow-up rate of participants in this cohort as of June 2019 was 88%).

### Risk factor ascertainment

The modifiable risk factors of interest were body mass index, diet quality, physical activity, alcohol consumption, and smoking. NHS II participants reported height and body weight at enrolment and biennially thereafter. Validity of the self-reported weight was confirmed against staff measured weight in a separate study (r=0.97).^25^ Women reported their diet every four years by completing a semiquantitative food frequency questionnaire with 130 items; they were asked how often on average they had consumed a specified amount of foods during the previous year (0- 6 times a day).^26^ The Alternate Healthy Eating Index score (AHEI, range 0-110) was derived from the food frequency questionnaires as a measure of adherence to a high quality diet.^27^ Because alcohol was a separate modifiable factor of interest, we removed it from the overall AHEI score (modified AHEI, range 0-100). 

Participants also reported the average time per week spent in various physical activities (of moderate or vigorous intensity in leisure time) in 1991, 1997, 2001, 2005, 2009, 2011, and 2013, from which weekly expenditure in metabolic equivalents (MET-h/week) was calculated for each type of activity and then summed up to derive total physical activity.^21^ Alcohol consumption was self-reported using the same food frequency questionnaires assessing diet; total alcohol consumption (g/day) was calculated by summing the reported intakes from alcoholic beverages (12.8 g for one 360 mL can of beer, 11.0 g for one 120 mL glass of wine, 14.0g for one standard serving of liquor) in conjunction with reported frequency for each beverage type.^28^ Cumulative average was calculated for the modified AHEI, physical activity, and alcohol to represent long term behaviours and reduce measurement error.^20^ ^21^ ^22^ Smoking status (current, past, never) and quantity (number of cigarettes/day if currently smoking) were reported on the biennial questionnaires.

### Outcome ascertainment

During each follow-up cycle, participants who reported type 2 diabetes diagnosed by a physician (in response to “have you had diabetes mellitus diagnosed?”) received a mailed supplemental questionnaire to report symptoms, diagnostic tests, and hypoglycaemic treatment to confirm self-reported diagnoses. Before 1997, according to the National Diabetes Data Group criteria, a self-reported case was considered confirmed if at least one of the following was reported on the supplementary questionnaire^29^: one or more classic symptoms (excessive thirst, polyuria, weight loss, hunger, pruritus, or coma) and elevated plasma glucose concentrations (fasting ≥7.8 mmol/L; random ≥11.1 mmol/L); at least two elevated concentrations of plasma glucose on different occasions (fasting ≥7.8 mmol/L; random ≥11.1 mmol/L; 2 hour oral glucose tolerance test ≥11.1 mmol/L); or treatment with insulin or any oral hypoglycaemic agents. The criteria for type 2 diabetes diagnosis changed in 1998, with a lower level of fasting plasma glucose ≥7.0 mmol/L being sufficient for diagnosis instead of ≥7.8 mmol/L according to American Diabetes Association criteria.^30^ High accuracy (98%) and low frequency of under-reporting (0.5%) were previously validated for self-reported diagnoses against medical records in a subset of participants.^31^

### Covariate ascertainment

At baseline and during follow-up, participants reported and biennially updated information on personal, lifestyle, and reproductive characteristics. Family history of diabetes in the first degree relatives (ie, parents and siblings) was assessed in 1989, 1997, 2001, and 2005.

### Genetic risk score ascertainment for type 2 diabetes

Genetic data were available from participants who underwent genome wide association study (GWAS) from the NHS II nested case-control studies for chronic diseases or participants who underwent genotyping of candidate single nucleotide polymorphisms (SNPs) of type 2 diabetes as part of the Diabetes and Women’s Health study. Details about the GWAS/genotyping and quality assurance have been published previously.^32^ We constructed an unweighted genetic risk score by selecting 59 SNPs associated with type 2 diabetes. We excluded non-white women to reduce population stratification and women who had a poor genetic sample quality (ie, >53 SNPs that failed genotyping). Of the 4275 women with a history of gestational diabetes mellitus, 1372 with high quality genetic data were included in the genetic risk score analysis. Baseline characteristics by status of genetic risk score availability (yes, no) were similar between the two groups. For those participants with missing values in some SNPs, the score was rescaled by dividing by the number of available SNPs and then multiplying by 59.^32^

### Statistical analysis

For each participant, we calculated person time from the return date of the first eligible questionnaire after gestational diabetes mellitus to the date of diagnosis of type 2 diabetes, death, or the end of follow-up (June 2019), whichever occurred first. For a given follow-up cycle, values from the previous cycle were carried forward if a modifiable risk factor was missing or if the participant reported implausible values on the food frequency questionnaires (ie, >70 items left blank or total energy intake <500 or >3500 kcal/day; 1 kcal=4.18 kJ).^20^ We stopped updating the risk factors when a participant reported incident cancer or cardiovascular disease during follow-up, since such diagnoses might modify lifestyle, potentially leading to reverse causation. For a given follow-up period, person time and cycle specific data were excluded from analyses when a participant reported being currently pregnant, since pregnancy probably influenced maternal body weight and lifestyle factors.

We first examined the five modifiable risk factors individually. For individual risk factors, body mass index was categorised as <23.0 (reference), 23.0-24.9, 25.0-29.9, and ≥30.0.^3^ Modified AHEI score and physical activity (MET-h/week) were both categorised by quintiles, with the lowest groups set as the reference. Alcohol consumption was categorised into never (reference), 0-4.9, 5.0-14.9, and ≥15.0 g/day. Smoking status was coded as never (reference), past, and current. Among current smokers, light (1-14 cigarettes/day) and heavy (≥15 cigarettes/day) smoking were previously assessed separately. We merged these two categories into one (ie, current) after observing similar risks of type 2 diabetes. 

We used Cox proportional hazards models to calculate adjusted hazard ratios and 95% confidence intervals; the time scale in the Cox models was time since diagnosis of gestational diabetes mellitus. Covariates adjusted in the models included age (months), calendar period (indicator variables), race (white, non-white), parity (1, 2, ≥3), age at first live birth (<30, ≥30 years), total duration of breastfeeding (never to <1, 1-6, 6-12, >12 months), oral contraceptive use (never, past, current), menopausal status (pre-menopausal, post-menopausal), and family history of diabetes (yes, no), with all of the risk factors adjusted simultaneously. Since body mass index could be a mediator of the association between the other modifiable risk factors and incident type 2 diabetes, we presented the association of each of these factors with and without adjustment for body mass index. All covariates except race were updated every 2-4 years in the statistical analysis.

We next examined the combined associations of modifiable risk factors in relation to the risk of type 2 diabetes. Based on results of individual risk factors and current evidence on the associations of interest, we defined the following as the optimal level for each factor: having a body mass index of <25.0,^2^ ^33^ engaging in ≥150 min/week of moderate intensity or ≥75 min/week of vigorous intensity physical activity (equivalent to 7.5 MET-h/week),^34^ high quality diet (top two groups of the modified AHEI (divided by quintiles)), moderate consumption of alcohol (5.0-14.9 g/day),^22^ ^35^ and not currently smoking (ie, never or past smoker).^3^ Number of the risk factors at the optimal level was modelled as a categorical variable (0 (reference), 1, 2, 3, 4, 5). Because the other risk factors are key determinants of body mass index (which itself is a risk factor for type 2 diabetes), we also examined the combined associations of these factors by removing the body mass index component.

We further evaluated whether status of obesity, family history, or genetic susceptibility of type 2 diabetes would modify the association of modifiable risk factors with type 2 diabetes by stratifying by body mass index at baseline (<25.0 or ≥25.0; using the other four modifiable factors only), family history of diabetes (yes or no), or genetic susceptibility (high or low, defined as above or below the median of the genetic risk score (68.0), respectively). In addition, to examine whether having optimal levels of modifiable factors would mitigate the underlying risk owing to family history or genetic susceptibility, we created joint categories of the risk factors and status of family history or genetic susceptibility of type 2 diabetes, with the high risk group (ie, with family history of diabetes or high genetic susceptibility and not having optimal levels for any of the risk factors) as the reference group.

Additional analyses comprised of excluding women with a body mass index at baseline of less than 18.5 (n=63) to reduce the potential for reverse causality (eg, low body mass index owing to pre-existing or undiagnosed chronic disease), examining the proportional hazards assumption for individual modifiable factors by stratifying time since diagnosis of gestational diabetes mellitus (ie, ≤ *v* >16.3 years (the median)), and additionally adjusting for household income self-reported in 2001 for potential residual confounding owing to socioeconomic status and adjusting for history of recurrence of gestational diabetes mellitus. We excluded participants with type 2 diabetes diagnoses within two years after the index pregnancy (n=12) to eliminate any unrecognised type 2 diabetes before the pregnancy. We also imputed missing values for risk factors before categorising them with multiple imputation approach.^36^ Lastly, we examined participant change in the number of optimal levels of modifiable factors from baseline to the most recent follow-up period in association with type 2 diabetes risk. All statistical analyses were performed with SAS software (version 9.3; SAS Institute). P<0.05 was considered statistically significant unless otherwise stated.

### Patient and public involvement

No participants were involved in setting the research question or the outcome measures, nor were they involved in the design and implementation of the study. We plan to disseminate these findings to participants in our yearly newsletter and to the general public in a press release.

## Results

With a median follow-up of 27.9 years (89 340 person years), we documented 924 incident cases of type 2 diabetes among 4275 women with a history of gestational diabetes mellitus. At baseline, women who had optimal levels of four or more modifiable factors were more likely to be older at first birth, report longer breastfeeding duration, and be pre-menopausal, but less likely to have a family history of diabetes (table 1).

**Table 1:** Baseline population characteristics according to number of optimal modifiable factors in women with a history of gestational diabetes mellitus, based on data from the Nurses’ Health Study II (n=4275)*

Characteristic	No of optimal modifiable factors†					
	0 (n=83)	1 (n=810)	2 (n=1470)	3 (n=1284)	4 (n=546)	5 (n=82)
Mean (SD) age (years)	37.1 (4.6)	37.4 (4.5)	37.1 (4.4)	36.8 (4.4)	37.4 (4.4)	37.6 (4.2)
Mean (SD) age (years) at first report of gestational diabetes mellitus	30.1 (5.0)	30.8 (5.3)	30.5 (5.2)	30.2 (5.3)	31 (5.0)	30.9 (4.3)
White participants	78 (94.0)	746 (92.1)	1331 (90.5)	1170 (91.1)	512 (93.8)	79 (96.3)
Family history of diabetes	24 (28.9)	273 (33.7)	448 (30.5)	352 (27.4)	141 (25.8)	17 (20.7)
Mean (SD) age at first birth (years)	26.3 (4.7)	27.5 (4.9)	27.5 (4.7)	27.5 (4.8)	28.4 (4.8)	29.5 (4.1)
Median (IQR) parity	2 (2-3)	2 (2-3)	2 (2-3)	2 (2-3)	2 (2-3)	2 (2-2)
Total duration of breastfeeding						
˂1 month	34 (41.0)	245 (30.2)	373 (25.4)	273 (21.3)	100 (18.3)	7 (8.5)
1-6 months	15 (18.1)	130 (16.0)	227 (15.4)	182 (14.2)	76 (13.9)	17 (20.7)
6-12 months	14 (16.9)	139 (17.2)	284 (19.3)	226 (17.6)	106 (19.4)	19 (23.2)
≥12 months	20 (24.1)	296 (36.5)	586 (39.9)	603 (47.0)	264 (48.4)	39 (47.6)
Oral contraceptive use						
Never	6 (7.2)	99 (12.2)	230 (15.6)	201 (15.7)	72 (13.2)	9 (11.0)
Past	73 (88.0)	655 (80.9)	1129 (76.8)	976 (76.0)	441 (80.8)	70 (85.4)
Current	4 (4.8)	56 (6.9)	111 (7.6)	107 (8.3)	33 (6.0)	3 (3.7)
Menopausal status						
Pre-menopausal	79 (95.2)	786 (97.0)	1428 (97.1)	1255 (97.7)	536 (98.2)	81 (98.8)
Post-menopausal	4 (4.8)	24 (3.0)	42 (2.9)	29 (2.3)	10 (1.8)	1 (1.2)
Mean (SD) body mass index	31.9 (5.0)	31.6 (6.9)	27.5 (6.0)	24.8 (4.9)	22.2 (2.5)	21.7 (1.8)
Modified AHEI score, mean (SD)‡	36 (6.3)	36.8 (7.1)	39.6 (8.2)	44.5 (9.6)	51.5 (8.3)	54.6 (7.0)
Total energy intake, mean (SD), kcal/day	1771 (460)	1899 (544)	1920 (554)	1872 (543)	1856 (529)	1905 (562)
Smoking status, No. (%)						
Never	0	500 (61.7)	1028 (69.9)	916 (71.3)	382 (70.0)	46 (56.1)
Past	0	152 (18.8)	294 (20.0)	318 (24.8)	159 (29.1)	36 (43.9)
Current	83 (100.0)	158 (19.5)	148 (10.1)	50 (3.9)	5 (0.9)	0 (0.0)
Mean (SD) duration of leisure time physical activity (MET-h/week)§	3.1 (2.1)	5 (8.5)	14.2 (19.4)	23.7 (24.4)	30.3 (31.8)	33.7 (27.1)
Median (IQR) alcohol intake (g/day)	0.6 (0-1.9)	0 (0-1.1)	0 (0-1.8)	0.9 (0-2.7)	1.8 (0-6.2)	8.7 (6.6-11.4)

Data are number (%) of participants unless stated otherwise. AHEI=Alternate Healthy Eating Index; MET=metabolic equivalent; IQR=interquartile range; SD=standard deviation.

* Baseline was defined as the first pregnancy questionnaire cycle after incident gestational diabetes mellitus during follow-up, and the questionnaire cycle in 1991 for prevalent gestational diabetes mellitus at the start of follow-up (in 1991).

† The optimal level of each factor was defined as follows: currently non-smoker (including never or past smoker); body mass index <25.0; top two groups of the modified AHEI score (divided by quintiles); ≥150 min/week of moderate intensity physical activity or ≥75 min/week of vigorous intensity physical activity; alcohol intake 5.0-14.9 g/day.

‡ AHEI score excluding the component of alcohol was used in the current analysis (possible score range 0-100).

§ MET values from moderate or vigorous intensity of leisure time activities were summed up to derive total physical activities; 7.5 MET-h/week is equivalent to 150 min/week of moderate intensity physical activity or 75 min/week of vigorous intensity physical activity.

### Individual modifiable risk factors and type 2 diabetes risk

When we examined the modifiable factors individually, adjusting for major demographic and health related factors, a higher body mass index was significantly associated with the risk of type 2 diabetes: compared with women with a body mass index <23.0, the adjusted hazard ratios were 2.74 (95% confidence interval 1.68 to 4.49), 5.72 (3.75 to 8.71), and 16.38 (10.87 to 24.67) for the groups with body mass indexes of 23.0-24.9, 25.0-29.9, and ≥30.0, respectively (supplementary table 1). Physical activity was inversely associated with the risk of type 2 diabetes in a dose dependent fashion (P_trend_<0.001). AHEI score was inversely associated with type 2 diabetes risk (P_trend_=0.05), but the association lost significance after further adjustment for body mass index. We observed a U shaped association for alcohol with type 2 diabetes risk, with the lowest risk observed in the group with moderate consumption (ie, 5.0-14.9 g/day; hazard ratio 0.50, 95% confidence interval 0.37 to 0.67) compared with never drinkers. For both physical activity and alcohol consumption, further adjustment for body mass index modestly attenuated the associations, but they remained significant. Smoking was not significantly related to the risk of type 2 diabetes.

### Modifiable risk factors in combination and type 2 diabetes risk

The number of optimal levels of modifiable risk factors was significantly and inversely related to type 2 diabetes risk (table 2). Compared with women who did not have optimal levels of any factors, the adjusted hazard ratios for women who had optimal levels of one, two, three, four, and five factors was 0.94 (95% confidence interval 0.59 to 1.49), 0.61 (0.38 to 0.96), 0.32 (0.20 to 0.51), 0.15 (0.09 to 0.26), and 0.08 (0.03 to 0.23), respectively (P_trend_<0.001). When we removed the body mass index component from the equation and separately adjusted for it, the inverse association remained significant (P_trend_<0.001); women who had optimal levels of the other four factors had a 65% and 49% lower risk of type 2 diabetes (hazard ratio 0.35 (95% confidence interval 0.19 to 0.62) and 0.51 (0.28 to 0.94)), without and with additional adjustment for body mass index, respectively (supplementary table 2).

**Table 2:** Risk of type 2 diabetes in women with a history of gestational diabetes mellitus by number of optimal modifiable factors, based on data from the Nurses’ Health Study II (n=4275)

No of optimal modifiable factors‡	No of cases/person years	Absolute risk (No of cases/1000 person years)	Hazard ratio (95% CI)	
			Model 1*	Model 2†
0	27/1349.3	20.0	1.00 (reference)	1.00 (reference)
1	310/15 895.7	19.5	0.96 (0.61 to 1.51)	0.94 (0.59 to 1.49)
2	361/28 558.3	12.6	0.61 (0.39 to 0.95)	0.61 (0.38 to 0.96)
3	175/26 671.0	6.6	0.31 (0.19 to 0.49)	0.32 (0.20 to 0.51)
4	46/13 883.3	3.3	0.15 (0.09 to 0.25)	0.15 (0.09 to 0.26)
5	5/2982.3	1.7	0.08 (0.03 to 0.22)	0.08 (0.03 to 0.23)
P_trend_§	—	—	<0.001	<0.001

CI=confidence interval.

* Model 1 was adjusted for age (months).

† Model 2 was adjusted for age, as well as race (white, non-white), parity (1, 2, ≥3), age at first live birth (<30, ≥30 years), total duration of breastfeeding (none to <1, 1-6, 6-12, >12 months), oral contraceptive use (never, former, current), menopausal status (pre-menopausal, post-menopausal), family history of diabetes in first degree relatives (yes, no), and total energy intake (divided by quartiles, kcal/day; 1 kcal=4.18 kJ).

‡ The optimal level of each factor was defined as follows: current non-smoker (including never or past smoker); body mass index <25.0; top two groups of the modified Alternate Healthy Eating Index score (divided by quintiles); ≥150 min/week of moderate intensity physical activity or ≥75 min/week of vigorous intensity physical activity; alcohol intake 5.0-14.9 g/day.

§ Number of optimal levels of modifiable risk factors was entered as a continuous variable into the model to estimate P value for trend.

### Modifiable risk factors, body mass index status, and type 2 diabetes risk

Body mass index status (<25.0 *v* ≥25.0) did not significantly modify the associations mentioned above (P_interaction_=0.94) (table 3). Even among 2227 women who were overweight or obese (body mass index ≥25.0; n=2227), compared with those who did not have optimal levels of any risk factors, the adjusted hazard ratios for women who had optimal levels of one, two, three, and four factors were 1.05 (95% confidence interval 0.62 to 1.78), 0.82 (0.48 to 1.38), 0.60 (0.34 to 1.05), and 0.40 (0.18 to 0.91), respectively (P_trend_<0.001).

**Table 3:** Risk of type 2 diabetes in women with a history of gestational diabetes mellitus by number of optimal modifiable factors excluding body mass index, stratified by baseline body mass index, Nurses’ Health Study II (n=4212)*

No of optimal modifiable factors excluding body mass index†	No of cases/person years	Absolute risk (No of cases/1000 years)	Hazard ratio (95% CI)		
			Model 1‡	Model 2§	Model 2+body mass index¶
**Body mass index <25.0 (n=1 985 196 type 2 diabetes events)**					
0	8/829.0	9.7	1.00 (reference)	1.00 (reference)	1.00 (reference)
1	47/9483.3	5.0	0.71 (0.27 to 1.88)	0.73 (0.25 to 2.08)	0.52 (0.17 to 1.60)
2	78/17 445.7	4.5	0.62 (0.24 to 1.56)	0.65 (0.23 to 1.79)	0.53 (0.18 to 1.57)
3	51/14 616.8	3.5	0.36 (0.14 to 0.93)	0.38 (0.13 to 1.07)	0.38 (0.12 to 1.13)
4	12/3819.3	3.1	0.38 (0.13 to 1.14)	0.46 (0.14 to 1.51)	0.58 (0.17 to 2.03)
P_trend_**	—	—	0.001	0.006	0.22
**Body mass index ≥25.0 (n=2 227 728 type 2 diabetes events)**					
0	22/1103.3	19.9	1.00 (reference)	1.00 (reference)	1.00 (reference)
1	277/12456.9	22.2	1.02 (0.61 to 1.71)	1.05 (0.62 to 1.78)	0.92 (0.53 to 1.58)
2	293/16571.2	17.7	0.79 (0.47 to 1.31)	0.82 (0.48 to 1.38)	0.84 (0.49 to 1.44)
3	120/10020.9	12.0	0.57 (0.33 to 0.98)	0.60 (0.34 to 1.05)	0.69 (0.39 to 1.22)
4	16/1474.6	10.9	0.37 (0.17 to 0.83)	0.40 (0.18 to 0.91)	0.52 (0.22 to 1.20)
P_trend_	—	—	<0.001	<0.001	0.01
P_interaction_ by body mass index††	0.94	—	—	—	—

CI=confidence interval.

* Women with baseline body mass index <18.5 (n=63) were excluded from the analysis.

† The optimal level of each factor was defined as follows: current non-smoker (including never or past smoker); top two groups of the modified Alternate Healthy Eating Index score (divided by quintile); ≥150 min/week of moderate intensity physical activity or ≥75 min/week of vigorous intensity physical activity; alcohol intake 5.0-14.9 g/day.

‡ Model 1 was adjusted for age (months).

§ Model 2 was adjusted for age, as well as for race (white, non-white), parity (1, 2, ≥3), age at first live birth (<30, ≥30 years), total duration of breastfeeding (none to <1, 1-6, 6-12, >12 months), oral contraceptive use (never, former, current), menopausal status (pre-menopausal, post-menopausal), family history of diabetes in first degree relatives (yes, no), and total energy intake (divided by quartiles, kcal/day; 1 kcal=4.18 kJ).

¶ Body mass index (continuous) reported concurrently with other risk factors of interest was additionally adjusted for in model 2.

** Number of optimal levels of modifiable risk factors was entered as a continuous variable into the model to estimate the P value for trend.

†† Test of interaction was assessed using the likelihood ratio test comparing two models with and without the interaction terms between number of the optimal risk factors and the stratified covariate.

### Modifiable risk factors, family history or genetic susceptibility, and type 2 diabetes risk

The inverse associations between the number of optimal modifiable factors and type 2 diabetes risk persisted regardless of family history of diabetes and genetic susceptibility (P_trend_<0.001; n=1372 for genetic risk score analysis; table 4). Among women with a family history of diabetes, compared with women who did not have optimal levels of any factors, the adjusted hazard ratios for those with optimal levels of one, two, three, four, and five factors were 0.96 (95% confidence interval 0.45 to 2.08), 0.60 (0.28 to 1.28), 0.37 (0.17 to 0.81), 0.16 (0.07 to 0.38), and 0.13 (0.04 to 0.48), respectively. Similarly, among women with a higher genetic susceptibility (ie, above the median of genetic risk score), the number of optimal levels of modifiable factors was significantly and inversely related to type 2 diabetes risk. Compared with women who had optimal levels of up to one factor, adjusted hazard ratios were 0.73 (95% confidence interval 0.39 to 1.35), 0.42 (0.21 to 0.82), and 0.11 (0.04 to 0.29) for women with optimal levels of two, three, and four factors, respectively. We found no type 2 diabetes events among those participants who had optimal levels of all five factors (zero cases in 677.4 person years).

**Table 4:** Risk of type 2 diabetes in women with a history of gestational diabetes mellitus by number of optimal modifiable factors, according to family history or genetic risk of type 2 diabetes, based on data from the Nurses’ Health Study II (family history of diabetes: n=4275; genetic risk: n=1372)

No of optimal modifiable factors*	With family history of diabetes or high GRS¶				Without family history of diabetes or low GRS		
	No of cases/person years	Absolute risk (cases/1000 years)	Adjusted hazard ratio (95% CI)†		No of cases/person years	Absolute risk (cases/1000 years)	Adjusted hazard ratio (95% CI)
**With *v* without family history of diabetes**							
0	13/540.2	24.1	1.00 (reference)		14/809.2	17.3	1.00 (reference)
1	182/7163.3	25.4	0.96 (0.45 to 2.08)		128/8732.4	14.7	1.08 (0.54 to 2.17)
2	198/12 828.1	15.4	0.60 (0.28 to 1.28)		163/15 730.2	10.4	0.65 (0.33 to 1.29)
3	112/12 214.0	9.2	0.37 (0.17 to 0.81)		63/14 457.0	4.4	0.29 (0.14 to 0.60)
4	28/6475.8	4.3	0.16 (0.07 to 0.38)		18/7407.5	2.4	0.17 (0.07 to 0.38)
5	4/1484.8	2.7	0.13 (0.04 to 0.48)		1/1497.4	0.7	0.05 (0.01 to 0.41)
P_trend_‡	—	—	<0.001		—	—	<0.001
P_interaction_ by family history of diabetes§	—	—	—		—	—	0.47
**GRS**							
0 or 1**	47/2339.7	20.1	1.00 (reference)		47/2735.2	17.2	1.00 (reference)
2	60/4398.2	13.6	0.73 (0.39 to 1.35)		51/4958.4	10.3	0.61 (0.31 to 1.21)
3	39/4938.2	7.9	0.42 (0.21 to 0.82)		38/4801.3	7.9	0.42 (0.20 to 0.88)
4	12/3124.6	3.8	0.11 (0.04 to 0.29)		6/2553.0	2.4	0.09 (0.02 to 0.42)
5	0/677.4	0	NA		0/708.3	0	NA
P_trend_	—	—	<0.001		—	—	<0.001
P_interaction_ by genetic susceptibility	—	—	—		—	—	0.38

CI=confidence interval; GRS=genetic risk score.

* The optimal level of each factor was defined as follows: current non-smoker (including never or past smoker); body mass index <25.0; top two groups of the modified Alternate Healthy Eating Index score (divided by quintile); ≥150 min/week of moderate intensity physical activity or ≥75 min/week of vigorous intensity physical activity; alcohol intake 5.0-14.9 g/day.

† Model was adjusted for age (months), as well as race (white, non-white), parity (1, 2, ≥3), age at first live birth (<30, ≥30 years), total duration of breastfeeding (none to <1, 1-6, 6-12, >12 months), oral contraceptive use (never, former, current), menopausal status (pre-menopausal, post-menopausal), and total energy intake (divided by quartiles, kcal/day; 1 kcal=4.18 kJ).

‡ Number of optimal levels of modifiable risk factors was entered as a continuous variable into the model to estimate P value for trend.

§ Test of interaction was assessed using the likelihood ratio test comparing two models with and without the interaction terms between number of the optimal risk factors and the stratified covariate.

¶ The GRS analysis was restricted to participants who were white and had available GRS with a high quality of genotyping (number of single nucleotide polymorphisms failed genotyping <53; 1372 participants with 300 cases of type 2 diabetes after 31 234.2 person-years of follow-up). Genetic risk was characterised by a GRS using 59 candidate single nucleotide polymorphisms associated with risk of type 2 diabetes. Median GRS value (68.0) was used as the cut-off threshold for categorising high versus low genetic risk. Range of GRS in the analysis sample was 33.7-85.4.

** Participants with no or one optimal risk factor were merged into one group to increase model stability, owing to a small number of participants in the group having no optimal risk factors.

**Fig 1:**
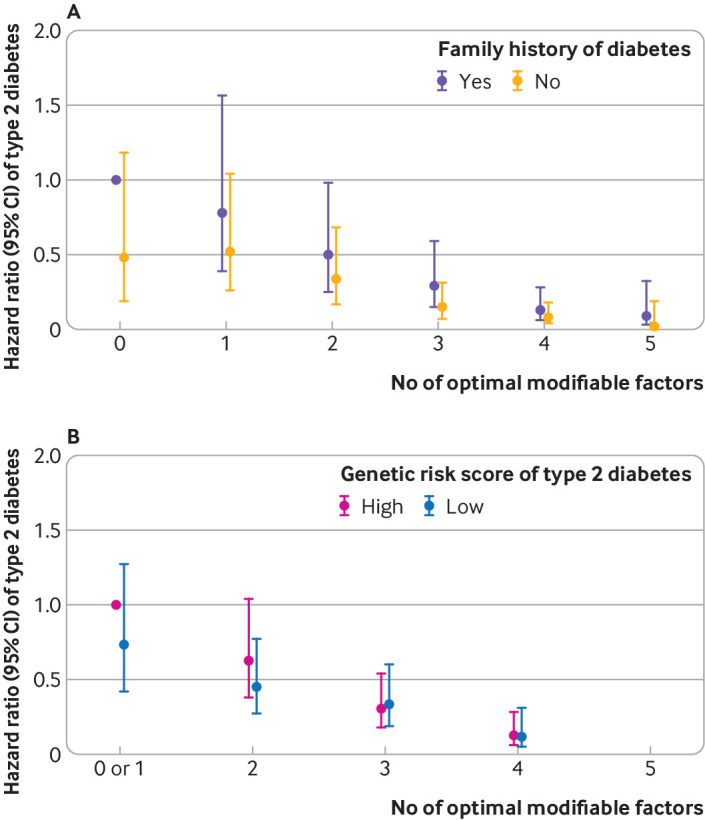
Risk of type 2 diabetes in women with a history of gestational diabetes mellitus by number of optimal modifiable factors, according to (A) family history of diabetes (yes *v* no) and (B) genetic risk score of type 2 diabetes (high *v* low (above *v* below median score of 68.0)) in the Nurses’ Health Study II. Joint categories of number of risk factors and covariate status were created, with the high risk group (ie, with family history of diabetes or high genetic risk score and having optimal level of zero or less than one factor set as the reference group depending on the model). CI=confidence interval

In the analyses on the joint categories of modifiable risk factors and status of family history or genetic susceptibility, while an elevated risk of type 2 diabetes was observed among women with a family history of diabetes or a greater genetic susceptibility, having a more favourable modifiable risk factor profile appeared to nearly eliminate the increased risk of type 2 diabetes associated with family history of diabetes or greater genetic susceptibility (fig 1).

Similar findings were observed in several sensitivity analyses. After excluding women with body mass index <18.5 (which could reflect low body weight owing to chronic illness), compared with the reference group (body mass index <23.0), the adjusted hazard ratios were 2.75 (95% confidence interval 1.67 to 4.52), 5.73 (3.73 to 8.80), and 16.45 (10.84 to 24.98) for body mass index groups 23.0-24.9, 25.0-29.9, and ≥30, respectively. When stratifying by the median time since diagnosis of gestational diabetes mellitus (≤ or >16.3 years), no violation of proportional hazards assumption was seen for any of the risk factors, and similar magnitudes of the associations were observed for the factors between the two time periods (results not shown). 

Additional adjustment for household income status or history of gestational diabetes mellitus recurrence or exclusion of type 2 diabetes diagnoses within two years after the index pregnancy did not influence the results (for having optimal levels of one, two, three, four, and five risk factors, respectively: adjusted hazard ratios 0.95 (95% confidence interval 0.60 to 1.51), 0.62 (0.39 to 0.98), 0.33 (0.20 to 0.52), 0.15 (0.09 to 0.26), and 0.09 (0.03 to 0.23 for income status; 0.94 (0.59 to 1.49), 0.61 (0.38 to 0.96), 0.32 (0.20 to 0.51), 0.15 (0.09 to 0.26), and 0.08 (0.03 to 0.23) for history of gestational diabetes mellitus recurrence; and 0.93 (0.58 to 1.48), 0.61 (0.38 to 0.96), 0.32 (0.20 to 0.51), 0.15 (0.09 to 0.25), and 0.08 (0.03 to 0.23) for exclusion of type 2 diabetes diagnoses); P_trend_<0.001). Results with imputed values for missing data of risk factors were consistent with the main results (supplementary table 3). In the analyses on change in lifestyle factors from baseline to the most recent follow-up, compared with women whose level of adherence remained stable, women who increased their number of optimal factors had a lower risk of type 2 diabetes. Conversely, we observed greater risk of type 2 diabetes among those who had a decrease in the number of optimal modifiable factors, after adjustment for baseline modifiable risk factors (supplementary table 4).

## Discussion

### Principal findings

In this large prospective cohort of high risk women with a history of gestational diabetes mellitus with 28 years of follow-up, having optimal levels of five modifiable risk factors was associated with a more than 90% relative reduction in the risk of incident type 2 diabetes after adjustment for other major diabetes risk factors. The beneficial associations were consistently seen, even among overweight/obese women and among women with greater genetic susceptibility to type 2 diabetes.

### Comparison with other studies and mechanistic insights

In the present study of predominantly white women with 28 years of follow-up and high retention rate, 21.6% women with a history of gestational diabetes mellitus (mean baseline age 37 years) developed type 2 diabetes. A recent meta-analysis observed a type 2 diabetes incidence rate of 16.2% by pooling seven cohorts of individuals with gestational diabetes mellitus and more than 10 years of follow-up.^17^ Differences in study exclusion criteria, follow-up length and retention rate, and racial or ethnic backgrounds might account for the different type 2 diabetes rates across studies. Prospective studies with long term follow-up on the association between combined modifiable risk factors and type 2 diabetes incidence among women with a history of gestational diabetes mellitus are limited. Inference from existing studies was hindered by relatively small sample size and short follow-up after the index pregnancy. 

Our findings are in general consistent with a subgroup analysis of women with a history of gestational diabetes mellitus (n=350) in the Diabetes Prevention Programme, where an intensive lifestyle intervention involving weight loss through diet and increased physical activity reduced the risk of type 2 diabetes by 50% after three years of follow-up compared with placebo.^37^ Updated findings from the main Diabetes Prevention Programme study supported the long term effectiveness of the lifestyle interventions on preventing type 2 diabetes over 15 years of follow-up.^38^ In the present study with 28 years’ follow-up among a larger number of women with a history of gestational diabetes mellitus, we showed a reduction of more than 90% in type 2 diabetes incidence associated with achieving optimal levels of five modifiable risk factors. In addition, our study confirmed and extended previous studies by showing that maintaining a normal weight and adopting healthy behaviours are independent of the underlying genetic risk and might even be able to fully offset a higher genetic risk of type 2 diabetes.^6^ ^7^

Despite the fact that our study cohort consisted entirely of registered nurses, less than 20% of the population reported having optimal levels of four or more risk factors over the follow-up, indicating the substantial public health opportunity to reduce type 2 diabetes among these high risk women. Notably, when we examined the change in adherence to optimal levels of modifiable factors from baseline to the most recent follow-up, improvement in the adherence was associated with a lower risk of type 2 diabetes, independent of baseline adherence levels. These findings suggest that these risk factors are modifiable and that improving adherence to optimal levels of modifiable factors later in life could have benefits in preventing type 2 diabetes.

A key message of the study was that for women with a history of gestational diabetes mellitus, incremental increase in the number of optimal modifiable factors was associated with a dose-dependent reduction of type 2 diabetes risk, even among those who were overweight or obese. Sustaining clinically significant weight loss is difficult^39^; for people who are already overweight or obese, such weight loss still might not reduce their risk to the level of women who maintained their body mass index in the normal range (ie, 18.5-24.9). In our study, while weight maintenance was important among those with a normal body mass index, additional adherence to optimal levels of diet and lifestyle factors could further lower incidence of type 2 diabetes. On the other hand, for women who were overweight or obese, even when optimal weight control (ie, body mass index <25.0) cannot be achieved, we showed that adopting the four remaining optimal modifiable factors (ie, regular physical activity, high quality diet, moderate alcohol consumption, and not smoking) was associated with a 60% reduction in the incidence of type 2 diabetes. Notably, the magnitude of risk reduction associated with the remaining four healthy lifestyle factors was comparable to the magnitude achieved in the intensive lifestyle interventions in the Diabetes Prevention Programme study, where weight loss and increased physical activity were the primary focus of the intervention.^37^

When examining the risk factors individually, we observed that body mass index had the strongest association with the risk of type 2 diabetes. Furthermore, adjustment for body mass index substantially attenuated associations of other lifestyle factors with risk of type 2 diabetes, which highlights the importance of maintaining a healthy weight after pregnancies complicated by gestational diabetes mellitus. As dietary patterns, physical activity, alcohol consumption, and smoking have been all related to an individual’s body mass index, associations of these factors with risk of type 2 diabetes could be mediated through their impacts on subsequent body mass index. In the present study, to avoid the adjustment for body mass index as a mediator, we adjusted for the body mass index measured concurrently with individual modifiable factors, not when measured later than these modifiable factors. 

Yet, the adjustment of body mass index could have represented a degree of over-adjustment when assessing the associations between the other modifiable factors with type 2 diabetes, owing to the strong correlations of body mass index over time. The precise degree by which this risk factor mediates these associations is outside of the scope of this current work but warrants further investigation. We noted that the association of the modified AHEI with type 2 diabetes risk became non-significant after adjusting for body mass index. This finding contrasts with those from a previous report from the NHS II,^20^ where AHEI was inversely associated with risk of type 2 diabetes among women with a history of gestational diabetes mellitus, irrespective of body mass index adjustment. 

These differences could be due to several potential reasons. Firstly, our study separately analysed the association between diet quality and alcohol consumption with type 2 diabetes, whereas in the previous report, moderate alcohol consumption was included as a metric for defining a healthy diet, as shown by the 2-3 times greater amount of daily alcohol consumed in the top and bottom groups of each of the dietary patterns. We demonstrated in our study that moderate alcohol consumption was associated with a lower risk of type 2 diabetes, so the previously observed relation between dietary patterns and type 2 diabetes might have been driven in part by higher alcohol consumption. When we conducted an analysis adding alcohol to the AHEI, we found a stronger association of AHEI than in our primary analysis that treated alcohol as a separate factor. With more years of follow-up, the mean AHEI score in our study (divided by quintile) had also increased to 34.0 for group 1 and 56.3 for group 4 compared with 25.5 for group 1 and 52.4 for group 4 in the previous analysis. The overall increase in diet quality in the cohort could have made it more difficult to detect a difference in the risk of type 2 diabetes attributable to diet between the lowest and highest groups in the present study.

Physical activity has been shown to be inversely related to type 2 diabetes risk in the general population.^40^ Among women with gestational diabetes mellitus, we observed that greater physical activity was independently associated with a lower risk of type 2 diabetes even after adjusting for body mass index. Independent of overall weight, physical activity can improve the ratio of lean to fat mass, improve insulin sensitivity, and reduce abdominal obesity.^41^ We did not find an association of current or former smoking with the risk of type 2 diabetes. A previous meta-analysis found that both current and former smokers had a modestly higher risk of developing type 2 diabetes.^42^ Compared with previous literature assessing smoking and type 2 diabetes in older female individuals, our null findings might be due to the overall moderate smoking behaviours in our study population, possibly an effect of their younger age.^3^ A complication of gestational diabetes mellitus as an early sign of metabolic abnormality might also have motivated these women to reduce their smoking intensity or to quit altogether. 

Alcohol consumption has a U shaped association with incident type 2 diabetes in the general population, with moderate consumption being associated with lower risk of type 2 diabetes, particularly among women,^22^ ^43^ whereas heavier consumption was associated with an increased risk. We were able to confirm this association in our study. Emerging studies suggest that even moderate intake of alcohol might be associated with higher risk of other diseases such as liver diseases, certain cancers, and possibly cardiovascular disease. The current dietary guidelines state that women who abstained from alcohol consumption should not start drinking for any reason. For current drinkers, drinking less is better for health than drinking more, and alcohol should be consumed in moderation (up to one serving/day for women).^35^ Owing to the timing of our data collection, we were not able to precisely assess whether the initial risk factor to self-reported alcohol consumption might have occurred during pregnancy. Pregnant individuals and those who are planning to become pregnant (given the frequent delay from initial conception to detection of pregnancy) might want to refrain from alcohol consumption given the known harms to the fetus (eg, growth deficiency and central nervous system impairments) irrespective of any potential metabolic benefits of moderate alcohol consumption.

### Strengths and limitations of the study

Our study has several strengths. We used data from a large prospective cohort with repeated measurements of health related and behavioural risk factors, which helps to better capture long term lifestyle habits and reduce measurement error and misclassification on the risk factors. In this well characterised cohort, we adjusted for major known demographic, reproductive, and medical risk factors for type 2 diabetes with updated time varying information. The large number of type 2 diabetes cases and long follow-up duration provided our study with good statistical precision to estimate the associations between both individual and combined modifiable factors and the disorder. With available genetic data, we were able to evaluate the associations of interest in conjunction with underlying genetic susceptibility of type 2 diabetes.

The study also has several limitations. Measurement error from self-reported modified risk factors and covariates was inevitable. However, owing to the prospective design, misclassification would probably not differ with respect to the type 2 diabetes outcome. Self-reported weight and lifestyle habits in NHS II have been validated in previous studies. The population studied consisted predominantly of healthcare professionals of European ancestry, which might limit the generalisability of our findings to individuals of other racial or ethnic groups or socioeconomic groups. However, the relative racial/ethnic and socioeconomic homogeneity in our population could help to reduce unmeasured confounding and improve internal validity, particularly related to access to healthcare, surveillance bias, and structural factors, and reduce error from self-reported health related behaviours. 

In stratified analyses by body mass index, subgroup analyses particularly among women with body mass index <25 and test of interactions were probably underpowered owing to the small number of type 2 diabetes events in this stratum. The NHS II ascertained physical activity levels from leisure time activities. Other activities, including transportation, occupation, and household activities, could contribute additional information to the role of total (work related and leisure time) physical activity for type 2 diabetes prevention, which warrants further investigations. Owing to the observational nature of the study, we cannot presume causality, though the risk factors we studied have been repeatedly shown in previous experimental studies to be associated with diabetes prevention. We did not have information on the severity of gestational diabetes mellitus or baseline glycaemic control, and whether these clinical factors could influence the associations of interest is subject to further investigation. Our genetic risk score only used 59 SNPs that have been previously shown to be associated with type 2 diabetes, and the use of a larger number of SNPs might enable future studies to stratify analysis into finer categories of genetic risk. Lastly, women who were aware of their heightened risk and consequently modified their risk factors might also have been more likely to get screened regularly for type 2 diabetes. Therefore, our methods may have underestimated the benefit associated with the optimal modifiable factors under study.

### Conclusions

In this prospective cohort study among women with a history of gestational diabetes mellitus with 28 years of follow-up, we identified an inverse association between the number of optimal modifiable risk factors and risk of incident type 2 diabetes. Participants who had optimal levels of all five modifiable factors after the index pregnancy had a more than 90% lower risk for developing type 2 diabetes compared with those who did not have any. Importantly, the lower risk of type 2 diabetes associated with optimal levels of modifiable risk factors was evident even among high risk women who were overweight or obese or who had higher genetic susceptibility.

**What is already known on this topic**

Adherence to optimal levels of modifiable risk factors (including weight control) such as following a healthy dietary pattern, engaging in regular physical activity, consuming alcohol in moderation, and avoiding smoking is associated with a lower risk of developing type 2 diabetes in generally healthy middle aged populations
Less is known about whether adherence to optimal levels of these modifiable risk factors is associated with a lower risk of developing type 2 diabetes in high risk women with a history of gestational diabetes and if obesity status or genetic risk of type 2 diabetes influences this association

**What this study adds**

In a large prospective cohort of women with a history of gestational diabetes with 28 years of follow-up, an inverse dose-response association was observed between the number of optimal modifiable risk factors and incidence of type 2 diabetes
The inverse association persisted among women who were overweight or obese, or were at greater genetic risk of type 2 diabetes
This study highlights the important public health opportunity for the prevention of type 2 diabetes in this high risk population

## Data Availability

Analytical code used for the present analysis might be made available on a case-by-case basis with approval from the senior author of this manuscript. Data described in the manuscript will not be made publicly available. Further information including the procedures for obtaining and accessing data from the Nurses’ Health Studies II is described online (https://www.nurseshealthstudy.org/researchers; email nhsaccess@channing.harvard.edu).

## Web extra

Extra material supplied by authors

Web appendix: Supplementary materials

## Notes

Contributors: JY and FQ served as co-first authors and contributed equally to this work. JY and FQ conducted the data analysis. JY and FQ drafted the first version of the manuscript. CZ, JEC, and FBH obtained funding. CZ designed, obtained funding, and provided oversight of the Diabetes and Women’s Health study. All authors contributed to the interpretation of the results and critical revision of the manuscript for important intellectual content and approved the final version of the manuscript. The corresponding author attests that all listed authors meet authorship criteria and no others meeting the criteria have been omitted. CZ is the study guarantor and accepts full responsibility for the work and the conduct of the study, had access to the data, and controlled the decision to publish.

The senior author (the manuscript’s guarantor) affirms that the manuscript is an honest, accurate, and transparent account of the study being reported; that no important aspects of the study have been omitted; and that any discrepancies from the study as planned (and, if relevant, registered) have been explained.

Dissemination to participants and related patient and public communities: For study participants, we will periodically update them on findings from the study through multiple channels, including our yearly newsletter, the study website, and our social media feeds. For the general public, we will disseminate findings through the media departments of the authors’ institute.

Provenance and peer review: Not commissioned; externally peer reviewed.
