# Supplementary material for: Modifiable risk factors and long term risk of type 2 diabetes among individuals with a history of gestational diabetes mellitus: prospective cohort study
Source: BMJ. 2022 Sep 21;378:e070312. doi: 10.1136/bmj-2022-070312 (PMC9490550; doi:10.1136/bmj-2022-070312)
Supplement: Supplementary file 1 — Web appendix: Supplementary materials [file yanj070312.ww.pdf]

Supplementary Table 1 Risk of type 2 diabetes associated with individual modifiable risk factors in women with a history of gestational diabetes mellitus, Nurses' Health Study II (n=4,275)

Risk factor	Case/ person-years	Absolute risk (case per 1,000 person-year)	Model 1[a] HR (95% CI)	Model 2[b] HR (95% CI)	Model 2 + BMI[c] HR (95% CI)
BMI, kg/m^2					
<23.0	26/21421.9	1.2	1.00 (ref)	1.00 (ref)	
23.0-24.9	49/13458.2	3.6	2.77 (1.70 to 4.51)	2.74 (1.68 to 4.49)	
25.0-29.9	208/27247.7	7.6	5.96 (3.93 to 9.03)	5.72 (3.75 to 8.71)	
30.0+	641/27212.0	23.6	18.53 (12.40 to 27.70)	16.38 (10.87 to 24.67)	
Physical activity (median, MET-hr/week)					
Q1 (1.0)	277/17584.2	15.8	1.00 (ref)	1.00 (ref)	1.00 (ref)
Q2 (5.1)	213/17693.2	12.0	0.75 (0.62 to 0.92)	0.76 (0.62 to 0.93)	0.92 (0.74 to 1.15)
Q3 (10.9)	174/17700.9	9.8	0.61 (0.49 to 0.75)	0.63 (0.50 to 0.76)	0.89 (0.71 to 1.12)
Q4 (20.9)	153/17988.7	8.5	0.53 (0.43 to 0.66)	0.58 (0.47 to 0.73)	0.85 (0.67 to 1.08)
Q5 (46.2)	107/18372.8	5.8	0.36 (0.28 to 0.46)	0.38 (0.29 to 0.49)	0.62 (0.48 to 0.81)
p-trend[d]			<0.001	<0.001	<0.001
Modified AHEI without alcohol (median)					
Q1 (32.9)	211/17317.5	12.2	1.00 (ref)	1.00 (ref)	1.00 (ref)
Q2 (39.7)	201/17504.3	11.5	0.84 (0.68 to 1.04)	0.94 (0.75 to 1.17)	1.01 (0.80 to 1.28)
Q3 (44.6)	196/17775.4	11.0	0.77 (0.62 to 0.96)	0.95 (0.76 to 1.19)	0.97 (0.77 to 1.23)
Q4 (49.8)	169/18067.7	9.4	0.64 (0.51 to 0.80)	0.86 (0.67 to 1.09)	0.90 (0.70 to 1.16)
Q5 (57.7)	147/18674.9	7.9	0.54 (0.42 to 0.68)	0.79 (0.61 to 1.01)	0.96 (0.73 to 1.25)
p-trend			<0.001	0.05	0.51

Alcohol, g/day					
Never	368/29775.6	12.4	1.00 (ref)	1.00 (ref)	1.00 (ref)
0-4.9	461/42805.9	10.8	0.82 (0.70 to 0.96)	0.89 (0.76 to 1.04)	0.97 (0.82 to 1.15)
5.0-14.9	71/13691.8	5.2	0.40 (0.30 to 0.53)	0.50 (0.37 to 0.67)	0.68 (0.50 to 0.92)
15.0+	24/3066.4	7.8	0.56 (0.36 to 0.88)	0.64 (0.39 to 1.03)	0.82 (0.50 to 1.34)
Smoking					
Never	628/60568.8	10.4	1.00 (ref)	1.00 (ref)	1.00 (ref)
Past	230/21968.9	10.5	0.98 (0.83 to 1.16)	1.05 (0.89 to 1.25)	0.91 (0.76 to 1.09)
Current	66/6802.0	9.7	0.92 (0.70 to 1.22)	0.87 (0.65 to 1.16)	0.99 (0.73 to 1.33)

Abbreviations: AHEI: Alternate Healthy Eating Index; BMI: body mass index; CI: confidence interval; GDM: gestational diabetes mellitus; HR: hazard ratio; T2D: type 2 diabetes.

[a] Model 1 was adjusted for age (months).

[b] Model 2 was additionally adjusted for race (white, non-white), parity (1, 2, 3+), age at first live birth (<30 years, ≥30 years), total duration of breastfeeding (none to < 1 month, 1-6 months, 6-12 months, >12 months), oral contraceptive use (never, past, current), menopausal status (premenopausal, postmenopausal), family history of diabetes in first-degree relatives (yes, no), total energy intake in kcal/day (quartiles), and all the other risk factors except BMI depending on the model (modified AHEI score in quintiles, physical activity in quartiles, categorical alcohol, categorical smoking status).

[c] BMI (kg/m^2, continuous) reported concurrent with other risk factors of interest was additionally adjusted.

[d] Number of optimal levels of modifiable risk factors was entered as a continuous variable into the model to estimate *p*-value for trend.

Supplementary Table 2 Risk of type 2 diabetes in women with a history of gestational diabetes mellitus by number of optimal modifiable factors excluding body mass index, Nurses' Health Study II (n=4,275)

	Cases/person-years	Absolute risk (case/1,000 person-year)	Model 1[a] HR (95% CI)	Model 2[b] HR (95% CI)	Model 2+BMI[c] HR (95% CI)
Number of optimal modifiable factors excluding BMI[d]					
0	30/1968.6	15.2	1.00 (ref)	1.00 (ref)	1.00 (ref)
1	324/22248.1	14.6	1.00 (0.65 to 1.52)	1.04 (0.68 to 1.60)	0.79 (0.50 to 1.24)
2	371/34478.0	10.8	0.71 (0.47 to 1.07)	0.75 (0.49 to 1.15)	0.73 (0.47 to 1.13)
3	171/25195.1	6.8	0.42 (0.27 to 0.65)	0.46 (0.29 to 0.72)	0.54 (0.34 to 0.87)
4	28/5450.0	5.1	0.31 (0.18 to 0.55)	0.35 (0.19 to 0.62)	0.51 (0.28 to 0.94)
p-trend[e]			<0.001	<0.001	<0.001

Abbreviations: AHEI: Alternate Healthy Eating Index; BMI: body mass index; CI: confidence interval; GDM: gestational diabetes mellitus; HR: hazard ratio; T2D: type 2 diabetes.

[a] Model was adjusted for age (months).

[b] Model was additionally adjusted for race (white, non-white), parity (1, 2, 3+), age at first live birth (<30 years, ≥30 years), total duration of breastfeeding (none to < 1 month, 1-6 months, 6-12 months, >12 months), oral contraceptive use (never, former, current), menopausal status (premenopausal, postmenopausal), family history of diabetes in first-degree relatives (yes, no), and total energy intake (quartiles, kcal/day).

[c] BMI (kg/m^2, continuous) reported concurrent with other risk factors of interest was additionally adjusted.

[d] The optimal level of each factor was defined as follows: 1) current non-smoker (including never or past smoker); 2) top two quintiles of the modified AHEI score; 3) ≥150 min/week of moderate-intensity or ≥75 min/week of vigorous-intensity physical activity; 4) alcohol intake 5.0-14.9 g/day.

[e] Number of optimal levels of modifiable risk factors was entered as a continuous variable into the model to estimate *p*-value for trend.

Supplementary Table 3 Risk of type 2 diabetes in women with a history of gestational diabetes mellitus by number of optimal modifiable factors using multiple imputation for modifiable factors with missing values, Nurses' Health Study II (n=4,275)

	Model 1[a] HR (95% CI)	Model 2[b] HR (95% CI)
Number of optimal modifiable factors[c]		
0	1.00 (ref)	1.00 (ref)
1	0.97 (0.56 to 1.68)	0.92 (0.54 to 1.58)
2	0.61 (0.35 to 1.08)	0.61 (0.35 to 1.08)
3	0.35 (0.20 to 0.60)	0.36 (0.21 to 0.62)
4	0.13 (0.07 to 0.23)	0.13 (0.07 to 0.24)
5	0.12 (0.05 to 0.30)	0.12 (0.05 to 0.29)
p-trend[d]	<0.001	<0.001

Abbreviations: AHEI: Alternate Healthy Eating Index; BMI: body mass index; CI: confidence interval; GDM: gestational diabetes mellitus; HR: hazard ratio; T2D: type 2 diabetes.

[a] Model was adjusted for age (months).

[b] Model was additionally adjusted for race (white, non-white), parity (1, 2, 3+), age at first live birth (<30 years, ≥30 years), total duration of breastfeeding (none to < 1 month, 1-6 months, 6-12 months, >12 months), oral contraceptive use (never, former, current), menopausal status (premenopausal, postmenopausal), family history of diabetes in first-degree relatives (yes, no), and total energy intake (quartiles, kcal/day).

[c] The optimal level of each factor was defined as follows: 1) current non-smoker (including never or past smoker); 2) BMI < 25.0 kg/m^2; 3) top two quintiles of the modified AHEI score; 4) ≥150 min/week of moderate-intensity or ≥75 min/week of vigorous-intensity physical activity; 5) alcohol intake 5.0-14.9 g/day.

[d] Number of optimal levels of modifiable risk factors was entered as a continuous variable into the model to estimate *p*-value for trend.

Supplementary Table 4 Change in the number of optimal levels of modifiable factors from baseline to the most recent follow-up in association with T2D risk in women with a history of gestational diabetes mellitus, Nurses' Health Study II (n=4,275)

	Cases/person-years	Absolute risk (case/1,000 person-year)	Model 1 HR (95% CI)[a]	Model 2 HR (95% CI)[b]	Model 2 + baseline number of optimal factors HR (95% CI)
Change in the number of optimal levels of modifiable factors[c]					
Decrease (-1 or more)	220/17168.4	12.8	0.99 (0.83 to 1.18)	0.96 (0.80 to 1.16)	1.58 (1.30 to 1.93)
No change (0)	450/32714.1	13.8	1.00 (ref)	1.00 (ref)	1.00 (ref)
Moderate increase (1)	202/26192.0	7.7	0.55 (0.46 to 0.66)	0.56 (0.46 to 0.67)	0.45 (0.37 to 0.55)
Great increase (2 or more)	52/13265.3	3.9	0.26 (0.19 to 0.36)	0.26 (0.19 to 0.36)	0.14 (0.10 to 0.20)

Abbreviations: AHEI: Alternate Healthy Eating Index; BMI: body mass index; CI: confidence interval; GDM: gestational diabetes mellitus; HR: hazard ratio; T2D: type 2 diabetes. ss

[a] Model was adjusted for age (months).

[b] Model was additionally adjusted for race (white, non-white), parity (1, 2, 3+), age at first live birth (<30 years, ≥30 years), total duration of breastfeeding (none to < 1 month, 1-6 months, 6-12 months, >12 months), oral contraceptive use (never, former, current), menopausal status (premenopausal, postmenopausal), family history of diabetes in first-degree relatives (yes, no), and total energy intake (quartiles, kcal/day) at baseline.

[c] The optimal level of each factor was defined as follows: 1) current non-smoker (including never or past smoker); 2) BMI < 25.0 kg/m^2; 3) top two quintiles of the modified AHEI score; 4) ≥150 min/week of moderate-intensity or ≥75 min/week of vigorous-intensity physical activity; 5) alcohol intake 5.0-14.9 g/day.
